# Toti-N-glycan Recognition Enables Universal Multiplexed Single-Nucleus RNA Sequencing

**DOI:** 10.34133/research.0678

**Published:** 2025-04-22

**Authors:** Yiran Guo, Liang Zhang, Xing Zhao, Chang Xu, Yiyang Li, Zhaolong Gao, Gaozhi Ou, Peng Chen, Wenshan Zheng, Hao Pei, Xin Liu, Bi-Feng Liu, Yiwei Li

**Affiliations:** ^1^ Key Laboratory of Molecular Biophysics of MOE and Hubei Bioinformatics & Molecular Imaging Key Laboratory, Department of Biomedical Engineering, College of Life Science and Technology - The Key Laboratory for Biomedical Photonics of MOE at Wuhan National Laboratory for Optoelectronics, Huazhong University of Science and Technology, Wuhan 430074, China.; ^2^ College of Sports Medicine, Wuhan Sports University, Wuhan 430079, China.; ^3^ John A. Paulson School of Engineering and Applied Sciences, Harvard University, Cambridge 021238, MA, USA.; ^4^ MobiDrop (Zhejiang), Tongxiang, Zhejiang 314500, China.; ^5^ School of Sports, China University of Geosciences, Wuhan 430074, China.

## Abstract

Sample barcoding-based multiplex single-cell and single-nucleus sequencing (sc/sn-seq) offers substantial advantages by reducing costs, minimizing batch effects, and identifying artifacts, thereby advancing biological and biomedical research. Despite these benefits, universal sample barcoding has been hindered by challenges such as inhomogeneous expression of tagged biomolecules, limited tagging affinity, and insufficient genetic insertion. To overcome these limitations, we developed Toti-N-Seq, a universal sample multiplex method, by tagging Toti-N-glycan on cell surfaces or nuclear membranes via our engineered streptavidin–Fbs1 GYR variant fusion protein, which could be used not only for sc-seq but also for sn-seq. Instead of targeting lipids or proteins, we focused on targeting the ubiquitous N-glycans found on any species with accessible membranes, which minimizes the exchange between barcoded samples and avoids biased barcoding. Our technology can be broadly applied to multiple species and nearly all eukaryotic cell types, with an overall classification accuracy of 0.969 for sc-seq and of 0.987 for sn-seq. As a demonstration with clinical human peripheral blood mononuclear cells, our Toti-N-Seq achieved rapid one-step sample preparation (<3 min) for easily scaling up while keeping high fidelity of sample ratios, removing artifacts, and detecting rare cell populations (~0.5%). Consequently, we offer a versatile platform suitable for various cell types and applications.

## Introduction

The dream of biologists to map the gene expression of every individual cell in a living organism has been partially realized through recent advances in single-cell technology [1–3]. The throughput of single-cell analysis has evolved from examining a single cell to hundreds of cells and now reaches a range of 10^3^ to 10^5^ cells or nuclei, thanks to technologies based on microwells, combinatorial indexing, and droplet microfluidics [4–9]. However, with approximately 36 trillion cells in a male body and 28 trillion in a female body, even descriptive analysis of living systems demands further scaling and substantially higher throughput [10].

The increasing demands from the pharmaceutical industry, clinical research, and developmental biology [11–14] have required single-cell and single-nucleus sequencing (sc/sn-seq) to be integrated with spatiotemporal information [15–19], combinational perturbations [20–22], patient identities [23,24], and experimental replicates [25,26] to minimize intersample variability and uncover correlations. The scaling of sc/sn-seq can be elegantly achieved by multiplexing samples prior to processing, utilizing barcoding through natural genetic variation, chemical labeling, oligonucleotide (oligo)-labeled antibodies, oligo-labeled lipid anchors, or genetic cell labeling [27–29]. Sample multiplexing involves labeling cells with sample-specific barcodes before pooling, allowing the sequencer to identify the origins of transcripts [30]. These methods have achieved increased throughput, eliminated batch effect, removed artifacts, and reduced costs [25,26]. Among these methods, genetic cell labeling suffers from varied efficiency between cell types and long-term preprocessing [27–29], which may also introduce “side-effect” perturbations similar to chemical labeling [25,27–29]. Antibody-based and lipid-based approaches are simple and generally applicable to a wide range of single-cell applications and platforms. However, the success of using antibodies for sample multiplexing depends on the ubiquitous expression of the target protein across cell types, which is challenging to be found to fit all nuclei and cell types, thus limiting the sample-agnostic universal application of multiplex sc/sn-seq [25,31–33]. In comparison, lipid-anchor-based methods can be widely applied across species and cell types, but they suffer weak and dynamic affinity between barcodes and lipids on cell/nucleus surfaces, which eventually lead to potential off-target effects and cross-contamination during preparation [26,33]. Overall, these methods tend to have a preference for either sc-seq or sn-seq [33].

**Fig. 1.**
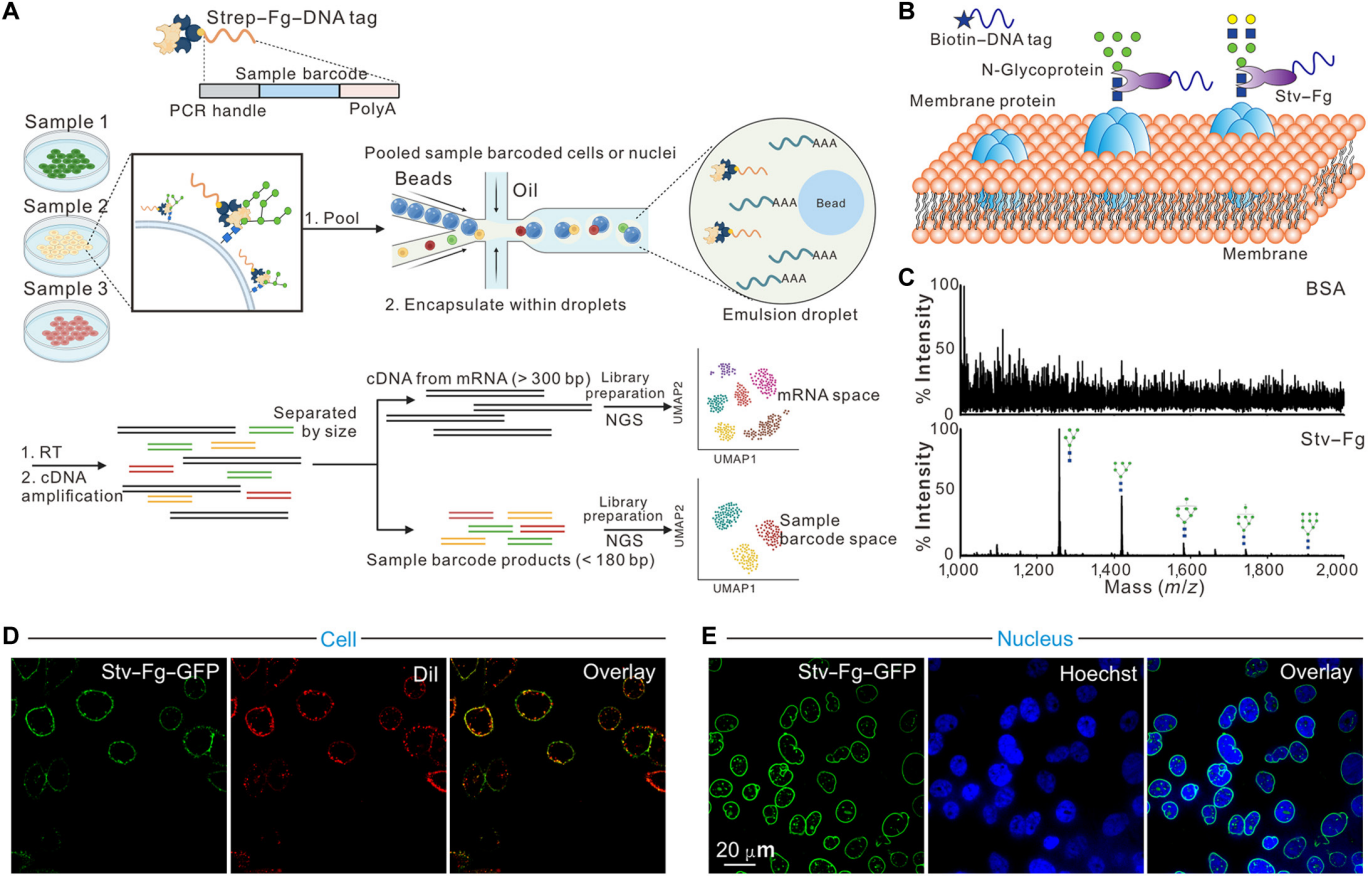
The concept of Toti-N-Seq for sample-multiplexed single-cell and single-nucleus sequencing (sc/sn-seq). (A) Schematic illustration of the workflow of Toti-N-Seq for sample-multiplexed sc/sn-seq. Created in BioRender. Zhao, X. (2025) https://BioRender.com/4n6oqs1. (B) Schematic illustration of engineered streptavidin–Fbs1 GYR variant (Stv–Fg) fusion protein for sample tagging. (C) Mass spectrometry showed the capability of engineered Stv–Fg for unbiased enrichment of N-glycan in cells. (D) Fluorescent imaging assays demonstrated the capability of engineered Stv–Fg fusion protein for unbiased and fluorescent cell tagging. (E) Fluorescent imaging assays demonstrated the capability of engineered Stv–Fg fusion protein for unbiased and fluorescent nucleus tagging. PCR, polymerase chain reaction; RT, reverse transcription; cDNA, complementary DNA; NGS, next-generation sequencing; mRNA, messenger RNA; BSA, bovine serum albumin; GFP, green fluorescent protein; Dil, 1,1′-dioctadecyl-3,3,3′,3′-tetramethylindocarbocyanine perchlorate.

To address these issues, we focus on tagging another biomolecule, N-glycan, which is universally presented on the surfaces of eukaryotic cells across various types and species [34–36]. N-glycans are a category of oligosaccharides that are covalently attached to proteins at asparagine (Asn) residues via an N-glycosidic bond [35]. They play a crucial role as information carriers on eukaryotic cell surfaces. There are many types of N-glycans [37]. Although the combination ratios of the types of N-glycans vary, the total amount of N-glycans remains roughly consistent [38]. Here, we engineered a Toti-N-glycan-recognizing protein with oligonucleotides that tags all types of N-glycans nonselectively, enabling universal sc-RNA sequencing (sc-RNA-seq) (Fig. 1A). Remarkably, our Toti-N-glycan barcodes are also capable of tagging nuclei; the sequencing assays confirm tight labeling on nuclei with an overall classification accuracy (OCA) of 0.987. Our Toti-N-glycan barcode further enables efficient removing of cell doublets, resulting in a doublet ratio below 0.04% for sc-seq and below 0.02% for sn-seq. With enhanced tagging efficiency and minimized crosstalk, our technology can preserve rare/minor subpopulations (~0.5% in population) within the multiplexed samples, allowing for high-throughput analysis without losing detailed cellular information in practical applications.

## Results

### Toti-N-Seq overview

Toti-N-Seq labeled cells or nuclei with DNA barcodes by recognizing the Toti-N-glycan on the membrane (Fig. 1A). N-Glycan associates with the membrane through covalently attaching to membrane proteins (Fig. 1B). These N-glycans, present on both nuclear and cell membranes, serve as anchors for DNA barcodes (Table S1), facilitating multiplex sequencing (multiplex-seq). To enable selective and universal tagging of N-glycan, we adapted a technology developed for N-glycan enrichment [39,40]. Our method takes advantage of the natural protein Fbs1, which functions within the ubiquitin-mediated degradation system to recognize the common core pentasaccharide motif (Man3GlcNAc2) of N-glycans [41]. Compared to wild-type Fbs1, the Fbs1 GYR variant (Fg) exhibits higher and unbiased recovery of all types of N-glycans [39,40]. To explore the Fg’s capability in cell/nucleus tagging, we engineered a streptavidin–Fg fusion protein (Stv–Fg), gaining more flexibility to link to biotin-modified groups, such as biotin–green fluorescent protein and biotin–oligonucleotide barcodes (Fig. 1B and Fig. S1). This allows us to link fluorescent barcodes or DNA barcodes to Fg via streptavidin, which then recognizes and labels all types of N-glycans on cell or nuclear membranes.

A Toti-N-Seq sample barcode includes an oligonucleotide sequence with a 3′ polyA capture sequence, a 6-bp sample index, and a 5′ polymerase chain reaction handle necessary for hybridization and library construction. After following the conventional steps of droplet-based sc/sn-seq, transcripts and Toti-N-Seq sample barcodes are linked to a common cell- or nucleus-specific barcode during reverse transcription, which enables sample demultiplexing. Toti-N-Seq sample barcodes and complementary DNA libraries are separated by size selection before library construction, enabling pooled sequencing at user-defined proportions (Fig. 1A).

### Biochemical evaluation of the tagging performance of Toti-N-glycan barcodes

We evaluated whether the Stv–Fg fusion protein labels intact N-glycans unbiasedly with minimal exchange between samples during sample preparation. First, N-glycans from standard glycoprotein (RNase B) were enriched using our engineering Stv–Fg fusion protein and analyzed via mass spectrometry. With our engineered Stv–Fg fusion protein, all 5 N-glycans from RNase B were accurately detected amid a complex background of biomacromolecules (Fig. 1C). The results demonstrated that our engineered Stv–Fg fusion protein could selectively and unbiasedly recognize N-glycans in a complex solution, which paves the way for labeling cells and nuclei for multiplex sample sequencing.

To further explore the labeling capability of our engineered Stv–Fg fusion protein in living cells and nuclei, we linked the engineered Stv–Fg fusion protein with biotin–green fluorescent protein to form a Toti-N-glycan fluorescent barcode and then incubated it with freshly isolated cells and nuclei (Fig. 1D and E). For cell membrane tagging, we compared the staining efficiency of our Toti-N-glycan fluorescent barcode with DiI (1,1′-dioctadecyl-3,3,3′,3′-tetramethylindocarbocyanine perchlorate, a dye that labels cell membranes by tagging lipids in red). The Toti-N-glycan fluorescent barcode clearly stained the cell membrane and colocalized with DiI at cell boundaries. Remarkably, the Toti-N-glycan fluorescent barcode was more efficient in labeling cell membranes than DiI in 2 aspects: the Toti-N-glycan fluorescent barcode uniformly labeled every single cell, while DiI did not accurately label the membranes of some cells (Fig. 1D); when we overlapped Toti-N-glycan fluorescent barcode labeling with DiI, DiI exhibited more labeling on intracellular lipid structures (Fig. 1D). For nucleus tagging, we co-stained isolated nuclei with both our Toti-N-glycan fluorescent barcode and Hoechst. The imaging results showed clear nuclear boundary structures and the encapsulation of Hoechst-labeled DNA inside the Toti-N-glycan fluorescent barcode-labeled nuclear membrane (Fig. 1E). Together, these results demonstrate the capability of our engineered Stv–Fg fusion protein to tag both cell membranes and nuclear membranes, allowing its application in imaging and beyond.

**Fig. 2.**
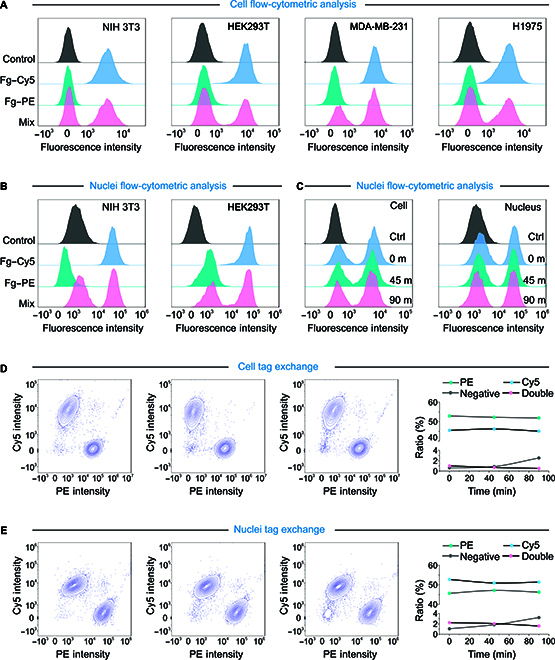
Evaluating the capability of the Toti-N-glycan barcode to demultiplex mixed samples using flow cytometry. (A) Two populations of cells were labeled by the Toti-N-glycan fluorescent barcode for different colors, respectively; each population of the cells maintained their tags after sample mixing. (B) Two populations of nuclei were labeled by the Toti-N-glycan fluorescent barcode for different colors, respectively; each population of the nuclei maintained their tags after sample mixing. (C) For different times of incubation, each population of cells and nuclei maintained their tags after sample mixing. (D) The Toti-N-glycan fluorescent barcode successfully demultiplexed tagged cells with minimized negatives and doublets. (E) The Toti-N-glycan fluorescent barcode successfully demultiplexed tagged nuclei with minimized negatives and doublets. Cy5, cyanine 5; PE, phycoerythrin.

Next, we used flow cytometry to evaluate whether our Toti-N-glycan fluorescent barcode could tag multiple cell types from different species. For 4 types of cells and nuclei from humans and mice—NIH 3T3 (mouse embryonic fibroblasts), HEK293T (human embryonic kidney cells), MDA-MB-231 (human breast adenocarcinoma cells), and H1975 (human non-small-cell lung cancer cells)—our Toti-N-glycan fluorescent barcode (Stv–Fg–Cy5 [cyanine 5] and Stv–Fg–PE [phycoerythrin]) successfully tagged all cell types (Fig. 2A and B). Additionally, we conducted a titration assay to determine the concentration required for efficient tagging of either cell membranes or nuclei (Fig. S2). By labeling cells and nuclei with varying concentrations of the Toti-N-glycan fluorescent barcode, we found that efficient labeling could be achieved at concentrations as low as 37.5 pM for cell membranes and 75.0 pM for nuclei (Fig. S2). Sample barcode exchange is one of the issues in multiplex sc/sn-seq. To test whether there is crosstalk after mixing 2 labeled populations of cells with dyes, we firstly incubated the mixed samples for different times and analyzed their signal afterward (Fig. 2C). The results showed that there was no obvious contamination between samples after mixing for up to 90 min (Fig. 2C). To further test whether our strategy minimizes exchange between samples, we mixed 2 labeled samples and use cell flow-cytometric analysis to separate them. The results showed that 2 populations of differentially labeled cells (Fig. 2D) or nuclei (Fig. 2E) could be easily separated by flow cytometry. We also compared the 2 populations with different incubation times (~0, 45, and 90 min) after mixing. For cell membrane tagging, within 45 min of incubation, only ~1% of cells were either negatively labeled or dual labeled (Fig. 2D). For nucleus tagging, even after 90 min of mixing, both the negatively labeled and dual-labeled populations remained below 2% (Fig. 2E). Since the cells’ encapsulation with compartments typically takes less than 15 min for various platforms [1,2], our Toti-N-glycan barcode provides sufficient high fidelity for tagging different samples during the library construction period.

### Toti-N-glycan barcodes for the sc/sn-seq of pooled samples

**Fig. 3.**
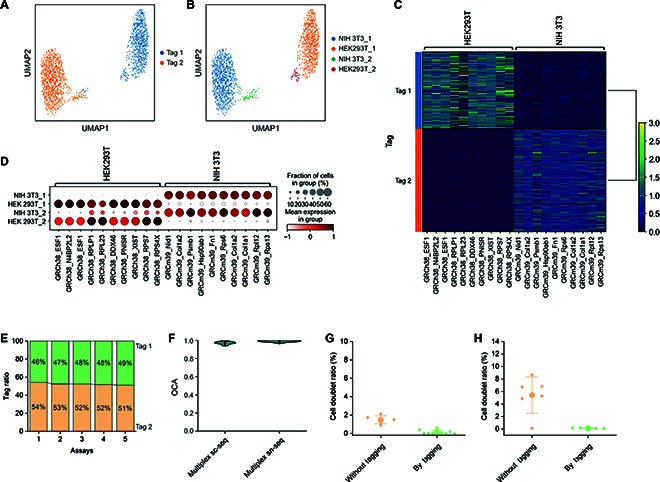
Toti-N-glycan barcodes for sn-seq of pooled samples. (A and B) Two sampled nuclei from 2 cell types were clustered based on their gene expression profiles. Each individual nucleus was indicated by their corresponding tags (A) and cell types (B). (C) Heatmap showing nuclei with different tags expressing different differentially expressed genes (DEGs). (D) Groups of nuclei exhibiting different DEGs. (E) In 5 parallel experiments, the ratio of 2 input samples detected by Toti-N-Seq, when the input ratio of 2 samples was 50 to 50. (F) Analysis of the overall classification accuracy (OCA) of our Toti-N-glycan-based multiplex sc-seq and sn-seq. (G) The ratio of doublets obtained by our Toti-N-multiplex sn-seq with or without sample tag detection. (H) The ratio of cell doublets obtained by our Toti-N-multiplex sc-seq with or without sample tag detection.

We validated the sample multiplexing capacity of Toti-N-Seq through proof-of-concept experiments using either whole cells or nuclei of HEK293T cells and NIH 3T3 cells (Fig. 3 and Fig. S3). Toti-N-glycan DNA sample barcodes were formed by linking biotinylated single-stranded DNA (biotin–ssDNA) to our Stv–Fg fusion protein. All samples were trypsinized, tagged with Toti-N-glycan DNA barcodes, and pooled before droplet encapsulation in a commercial droplet-based microfluidic device (MobiNova).

After sequencing, we analyzed a final sc/sn-RNA-seq dataset containing 80,000 nuclei (Fig. 3) and 100,000 cells (Fig. S3). We identified clusters in gene expression space according to different genes for HEK293T cells as well as for NIH 3T3 cells (Fig. 3B). Projection of Toti-N-glycan DNA sample barcode classifications onto gene expression space for the 2 sampled cell types revealed that the Toti-N-glycan DNA sample barcode successfully demultiplexed each sample (Fig. 3A and B). Indeed, when comparing the signature differentially expressed genes (DEGs) of NIH 3T3 cells and HEK293T cells, we found that the Tag 1 demultiplexed cells expressed only DEGs associated with HEK293T cells, while Tag 2 demultiplexed cells expressed only DEGs associated with NIH 3T3 cells (Fig. 3C). Clustering the cells by their signature DEGs efficiently grouped them into 2 clusters of HEK293T cells and 2 clusters of NIH 3T3 cells (Fig. 3D).

The ability of our technology to maintain the ratio of mixed input samples is crucial for distributing sequencing depth evenly across each sample. Therefore, we validated the fidelity of our technology in preserving the ratio of mixed input samples. In 5 parallel experiments, we pooled 2 different cell samples in a 1:1 ratio (HEK293T cells with NIH 3T3 cells, HEK293T cells with Michigan Cancer Foundation 7 [MCF7] cells, NIH 3T3 cells with MDA-MB-231 cells, HEK293T cells with peripheral blood mononuclear cells [PBMCs], and NIH 3T3 cells with H1975 cells). The deviation of input samples in the mixed ratio was consistently below 4%, ensuring balanced sequencing depth across various cell types (Fig. 3E). Overall, our technology demonstrates excellent fidelity when pooling samples, suggesting its potential for scaling up.

We next explored the potential mislabeling between 2 Toti-N-glycan DNA sample barcodes during multiplex-seq. To demultiplex sc/sn-RNA-seq, we predefined a confidence ratio of 0.9. To evaluate the capability of our technology to demultiplex mixed samples, we employed OCA. OCA is typically calculated as the overlap between cell line annotations produced by bioinformatic pipelines, such as Seurat, and freemuxlet as a reference annotation [42]. Specifically, OCA is defined as the number of all matching singlets plus the number of matching nonsinglets (mostly multiplets) divided by the total number of cells. To minimize potential technical noise from computational tools in identifying cell types, we used mixed samples of mouse and human cells. Whether the demultiplexed cells were correctly matched was assessed by determining whether the cells were correctly assigned to their original types based on the expression of mouse or human genes. As a result, our analysis revealed an OCA of 0.969 for sc-seq and of 0.987 for sn-seq, which surpassed the performance of either protein-tagged or lipid-tagged multiplex sc/sn-seq [33]. These results suggest that our method can accurately identify sample populations, outperforming other commercial multiplex sc-seq technologies that are based on either lipids or antibodies (Fig. 3F and Table S2 ) [33].

Moreover, the Toti-N-glycan sample barcode enables multiplex sc/sn-RNA-seq to identify cell/nucleus doublets. To assess possible cell/nucleus doublets, we grouped Toti-N-glycan multiplex-seq classifications according to cell-type composition. Initially, we identified cell doublets by the genes of HEK293T cells and NIH 3T3 cells. The analysis detected cell doublets in sc-seq at a ratio of approximately 5.42% (Fig. 3G) and nucleus doublets in sn-seq at a ratio of approximately 1.49% (Fig. 3H). In the second step, the sc-seq data were further filtered using Toti-N-glycan DNA sample barcodes. After removing doublets detected by our sample barcodes, to further verify the possible remaining doublets, we employed another widely used computational tool, Scrublet [43], to detect doublets and verify the capability of our barcode to exclude artifacts. As a result, the doublet ratios were computationally assessed to be 0.04% for sc-seq and 0.02% for sn-seq. Overall, the capability of our technology to identify artifacts and improve data quality underscores its universal applicability and scalability.

### Multiplexed sn-RNA-seq

**Fig. 4.**
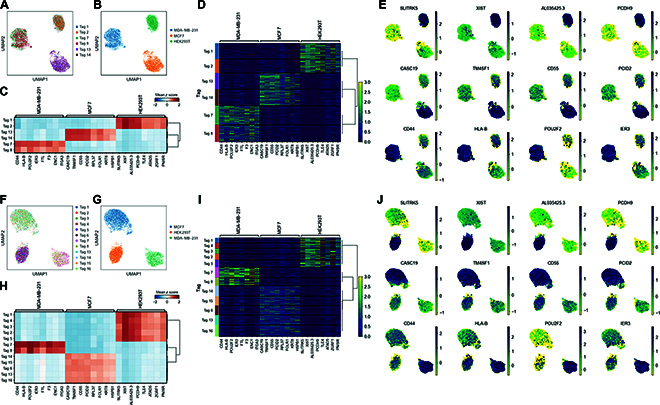
Six-plexed and 12-plexed sn-seq. (A and B) Six sampled nuclei from 3 cell types were clustered based on their gene expression profiles. Each individual nucleus was indicated by their corresponding tags (A) and cell types (B). (C) Groups of nuclei from 6-plexed samples exhibiting different DEGs. (D) Heatmap showing nuclei with different tags expressing different DEGs. (E) Expression of the signature DEGs of individual nuclei in gene expression space. (F and G) Twelve sampled nuclei from 3 cell types were clustered based on their gene expression profiles. Each individual nucleus was indicated by its corresponding tag (F) and cell type (G). (H) Groups of nuclei from 12-plexed samples exhibiting different DEGs. (I) Heatmap showing nuclei with different tags expressing different DEGs. (J) Expression of signature DEGs of individual nuclei in gene expression space.

Moreover, we further tested the scalability of our Toti-N-glycan multiplex sc/sn-seq with both homotypic and heterotypic populations. Given that the total sequencing depth depends on the sequencer’s capacity and the need to profile enough cells within a population to preserve heterogeneity and rare subpopulations, we argue that 6-plexed or 12-plexed sn-seq can meet most requirements of multiplex assays. For 6-plexed sn-seq, we pooled 2 samples of HEK293T cells, 2 samples of MDA-MB-231 cells, and 2 samples of MCF7 cells to evaluate the accuracy and fidelity of sample tagging (Fig. 4A to E). For the 12-plexed sn-seq, we pooled 5 samples of HEK293T cells, 2 samples of MDA-MB-231 cells, and 5 samples of MCF7 cells to evaluate the accuracy and fidelity of sample tagging (Fig. 4F to J and Fig. S4).

The 3 types of nuclei were well separated and clustered by their gene expression matrix and signature DEGs, which were conserved by our Toti-N-glycan barcodes (Fig. 4A, B, F, and G). For the 6-plexed sn-seq, only Tag 1 and Tag 2 were enriched in HEK293T cells, only Tag 13 and Tag 14 were enriched in MCF7 cells, and only Tag 7 and Tag 8 were enriched in MDA-MB-231 cells. No obvious cross-labeling was observed (Fig. 4A and B). For the 12-plexed sn-seq, only Tag 1, Tag 2, Tag 3, Tag 4, and Tag 5 were enriched in HEK293T cells; only Tag 7 and Tag 8 were enriched in MCF7 cells; and only Tag 6, Tag 13, Tag 14, Tag 15, and Tag 16 were enriched in MDA-MB-231 cells. Again, no obvious cross-labeling was observed (Fig. 4F and G). When we plotted the DEGs in each tag group, the signature DEGs (SLITRK5, XIST, AL035425.3, PCDH9, TLE4, ATAD5, ZGRF1, and PNISR) of HEK293T cells were highly expressed in Tag 1 and Tag 2 groups, the signature DEGs (CASC19, TM4SF1, CD55, PCID2, RPL37, FOLR1, KRT8, and HSPB1) of MCF7 cells [43,44] were highly expressed in Tag 13 and Tag 14 groups, and the signature DEGs (CD44, HLA-B, POU2F2, IER3, FTL, F3, ENC1, and ITGA3) of MDA-MB-231 cells [43,44] were highly expressed in Tag 7 and Tag 8 groups (Fig. 4C). A consistent enrichment was also observed in the 12-plexed sn-seq (Fig. 4H). Additionally, we plotted global heatmaps of signature DEGs from both 6-plexed (Fig. 4D) and 12-plexed (Fig. 4I) sn-seq. The results showed that each group labeled by different tags exhibited similar amounts of sequenced nuclei, indicating high fidelity in tagging different samples as the multiplex sn-seq is scaled up. Thus, we argue that our technology enables even sequencing across samples while preserving their attributes. By analyzing DEGs among the subclusters, we consistently observed that the signature DEGs associated with cell identities were selectively distributed among subclusters (Fig. 4E and J).

### Demultiplexing PBMC subpopulations by Toti-N-glycan barcodes

For applications in animal studies or clinical research, samples usually contain multiple types of cells with unbalanced population distributions. Therefore, it is essential that our Toti-N-glycan barcode can preserve minor subpopulations within the sample and maintain their population information. To evaluate this, we aimed to demultiplex PBMCs from humans. PBMCs, isolated from peripheral blood, are identified as blood cells with a round nucleus. These cells primarily include lymphocytes (T cells, B cells, and natural killer [NK] cells), monocytes, and dendritic cells. In most cases, lymphocytes constitute 70% to 90% of PBMCs (50% to 85% T cells, 5% to 10% B cells, and 5% to 20% NK cells), monocytes make up 5% to 20%, while dendritic cells (DCs) are rare, accounting for only about 2% [45]. Among the minor population of DCs, plasmacytoid dendritic cells (pDCs) are a unique subset specialized in secreting high levels of type I interferons, and they comprise only 0.2% to 0.8% of human PBMCs [46,47]. If Toti-N-glycan-guided multiplex sn-seq can reliably detect pDCs during demultiplexing, our technology can be confirmed as reliable for detecting rare cells in animal and clinical applications.

**Fig. 5.**
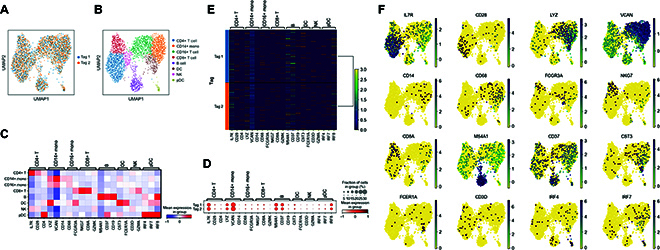
Toti-N-sn-seq demultiplexed pooled human peripheral blood mononuclear cell (PBMCs) while preserving minor/rare cell populations. (A and B) Two sampled nuclei from human PBMCs were clustered based on their gene expression profiles. Each individual nucleus was indicated by its corresponding tag (A) and cell type (B). (C) Groups of nuclei from different cell types in PBMCs exhibit different signature marker genes. (D) Two tagged samples of nuclei exhibit different signature marker genes. (E) Heatmap showing nuclei with different tags expressing different signature marker genes. (F) Expression of signature marker genes of individual nuclei from PBMCs in gene expression space. DC, dendritic cell; NK, natural killer; pDC, plasmacytoid dendritic cell.

In the plotted gene expression space of PBMCs from pooled human samples, we obtained well-categorized populations of cells that closely followed the expected cell types by their reported marker genes [48–51] (Fig. 5A and B), including CD14+ monocytes, CD16+ monocytes, CD4+ T cells, CD8+ T cells, B cells, NK cells, DCs, and pDCs. Within each population, our Toti-N-glycan tags were evenly distributed (Fig. 5A and B), suggesting that each tag unbiasedly labeled different cell types. By analyzing the expression of signature genes from different cell types, for different cell types, the subpopulations showed distinct patterns of signature gene expression (Fig. 5C). For different tagged groups, the nuclei labeled by Tag 1 and Tag 2 exhibited similar patterns (Fig. 5D). When we presented the gene expression heatmap with sample tags (Fig. 5E), we found that the proportion of each sample was 52.3% and 47.7% for Tag 1 and Tag 2, respectively, closely matching the input ratio of 50 to 50. Examining DEGs across subclusters, we found that cell-identity-associated signature genes exhibited distinct distribution patterns (Fig. 5F). Additionally, the cell doublet ratio was detected to be 0.6%, highlighting our technology’s capability to exclude artifacts. In our multiplex sn-seq of human PBMCs, the ratio of B cells ranged from 7.5% to 10%, the ratio of monocytes ranged from 27% to 42%, the ratio of NK cells ranged from 4.2% to 10%, and the ratio of T cells ranged from 47% to 51%. These detected ratios matched the reported ranges of cell types in human PBMCs as mentioned above [45–47]. Importantly, we successfully detected the ratio of DCs to be 2.5% to 4.4% and the ratio of pDCs to be 0.5% to 1.3%. These results suggest that our technology successfully detects rare cell types within pooled samples, with ratios as low as 0.5%. In addition to multiplexing human PBMCs, our technology was also demonstrated to multiplex mouse PBMCs (Fig. S5), as well as mixed human and mouse samples (Fig. S6). Together, our technology can multiplex samples with mixed populations across different species and obtain rare cell information, making it suitable for a wide range of applications in animal and clinical samples.

## Conclusion

Our technology leverages advancements in chemical biology for novel glycomics probes, which could be used not only for fluorescence microscopy imaging and flow cytometry analysis but also for sc/sn sequencing. By uniquely tagging global N-glycans on cell or nuclear surfaces, we enable multiplex sc/sn-seq. This approach offers distinct advantages over current protein- and lipid-based technologies. First, our Toti-N-glycan barcode can be used unbiasedly for both sc-seq and sn-seq, which is not achievable with either protein-based or lipid-based methods [33]. Additionally, the lack of bias across cell types and species makes our technology universally applicable to diverse samples. Its unbiased nature allows for accurate barcoding of complex cell-type mixtures in clinical samples, maintaining their original ratios. Furthermore, our Toti-N-glycan multiplex sc/sn-seq easily integrates with other biomolecular tags and genetic perturbations. Combining antibody or epigenetic tags with our technology enables multiplex sc/sn multi-omics sequencing, and integration with genetic editing facilitates multiplex perturb-seq for screening applications.

In conclusion, this study introduces a rapid and universal multiplexing technology for sc- and sn-RN-seq, substantially improving accuracy, efficiency, and scalability. By enabling the detection of rare cell types and minimizing batch effects, this method enhances data reliability and accessibility, facilitating advanced research in various biomedical fields. It supports large-scale studies, multi-omics integration, and translational applications, driving progress in both basic and clinical research. Consequently, our technology provides more opportunities for the translational applications of sc/sn-seq in fundamental research, clinical settings, and industrial uses. With unbiased tagging during sample pooling, this technology can be easily integrated with spatiotemporal information, combinational perturbations, patient identities, and experimental replicates, enabling multifaceted and functional sc/sn-seq for a wide range of applications.

## Methods

### Expression and purification of protein

Template DNA for Stv and Fg was amplified and cloned into a pET-28a vector, resulting in plasmids encoding Fg proteins with an N-terminal Stv tag (referred to as Stv–Fg). These plasmids were then transformed into *Escherichia coli* BL21 (DE3) competent cells for protein expression. The bacterial cells were cultured in Luria–Bertani medium containing 0.05 mg/ml kanamycin and induced with 0.1 mM isopropyl β-D-1-thiogalactopyranoside at 250 rpm and 16 °C for 16 h. Following induction, the cells were centrifuged at 8,000 rpm and 4 °C for 10 min. The resulting bacterial pellet was resuspended and disrupted using a high-pressure homogenizer to release the fusion proteins. The proteins were purified using Ni-Charged Resin, as described in our previous work, and then diluted in phosphate-buffered saline (PBS) for storage.

### Analytical flow cytometry

Analytical flow cytometry was performed using CytoFLEX (Beckman Coulter) to assess the labeling efficiency and membrane residency dynamics of Stv–Fg across various live-cell and nuclear membranes. The cell lines and nuclei used in these experiments included NIH 3T3, HEK293T, MCF7, and H1975. Throughout the experiments, the cells were maintained on ice. Sample preparation followed the same workflow as for the subsequent single-cell RNA sequencing and single-nucleus RNA sequencing experiments (see the “Cell barcoding” and “Nucleus barcoding” sections), with the key difference being that Stv–Fg was pre-incubated with biotin–Cy5 or biotin–PE at double the molar concentration, resulting in Stv–Fg with altered fluorescence properties (refer to the “Fluorescent Stv-Fg preparation” section in Supplementary Methods for details). Cy5 was excited at 638 nm and detected at 660 nm, while PE was excited at 561 nm and detected at 520 nm. Data acquisition was carried out using the CytExpert software (https://www.beckman.com/flow-cytometry/software), and FlowJo software (https://www.flowjo.com/) was used for data analysis and generating flow cytometry plots.

### Toti-N-glycan barcode preparation

A 100 μM solution of biotin-modified oligonucleotide barcode was first prepared according to the manufacturer’s instructions. Then, 5 μl of this solution was mixed with 30 μl of PBS buffer to achieve a concentration of 14.2 μM. Subsequently, 5 μl of the 14.2 μM oligo solution was combined with 5 μl of the 6.25 μM Stv–Fg solution and 90 μl of PBS, resulting in a final Toti-N-glycan barcode solution with an approximate concentration of 6.25 μM. Unless otherwise specified, all subsequent operations were performed using Sarstedt low-adhesion centrifuge tubes.

### Nucleus barcoding

Cell cultures or tissue samples were prepared, targeting a maximum of 500,000 cells per labeling condition. Cells were washed twice with PBS and dissociated enzymatically or mechanically to generate single-cell suspensions. Nuclei were isolated, after which the suspension was centrifuged (500 × g, 5 min) to pellet nuclei. All subsequent centrifugation steps adhered to this speed. Procedures and reagents were maintained on ice to preserve sample integrity. Following isolation, nuclei were washed twice with ice-cold PBS. For each wash, the pellet was resuspended and centrifuged (5 min, 4 °C), and the supernatant was carefully removed. A 100-μl aliquot of Toti-N-glycan barcode solution was then added to the nucleus pellet and incubated on ice for 5 to 30 min. Post-incubation, nuclei were washed with 1.9 ml of PBS and centrifuged (5 min, 4 °C), and the supernatant was discarded. This wash cycle—resuspension in 2 ml of PBS, centrifugation, and supernatant removal—was repeated twice to eliminate residual unbound barcodes. Finally, nucleus viability and concentration were assessed using acridine orange/propidium iodide dual-fluorescence staining and quantified with a Countstar Mira FL fluorescence cell analyzer. Processed samples were stored on ice until further use.

### Cell barcoding

Cell cultures or tissue samples were prepared identically to the nucleus barcoding protocol, targeting ≤500,000 cells per condition. Following dissociation into single-cell suspensions (as described above), cells—rather than nuclei—were centrifuged (400 × g, 5 min), with this speed maintained for subsequent steps. All procedures and reagents were kept on ice. After supernatant removal, 100 μl of Toti-N-glycan barcode solution was added to the cell pellet and incubated on ice for 5 to 30 min. Post-incubation, cells were washed twice with PBS (1.9 ml followed by 2 ml, centrifuged at 400 × g, 5 min, 4 °C), with supernatants discarded after each cycle. 

## Data Availability

All data are available upon reasonable request.

## Supplementary Materials

Supplementary 1 Supplementary Resources Table
