# Supplementary material for: Toti-N-glycan Recognition Enables Universal Multiplexed Single-Nucleus RNA Sequencing
Source: Research (Wash D C). 2025 Apr 22;8:0678. doi: 10.34133/research.0678 (PMC12348056; doi:10.34133/research.0678)
Supplement: Supplementary 1 — Supplementary Resources Table [file research.0678.f1.docx]

Supplementary Materials for

Toti-N-glycan recognition enables universal multiplexed

single nucleus RNA sequencing

Yiran Guo^1†^, Liang Zhang^2†^, Xing Zhao^3†^, Chang Xu^4†^, Yiyang Li^4^, Zhaolong Gao^1^, Gaozhi Ou^5^, Peng Chen^1^, Wenshan Zheng^4^, Hao Pei^4^*, Xin Liu^1^*, Bi-Feng Liu^1^*, Yiwei Li^1^*

†These authors contribute equally to this manuscript

*Correspondence to: yiweili@hust.edu.cn (Y.L.); bfliu@mail.hust.edu.cn (B.-F.L.); xliu@mail.hust.edu.cn (X.L.); haopei@mobidrop.com (H.P.)

**This PDF file includes:**

Resource table

Materials and Methods

Table S1 and S2

Figs. S1 to S6

References

**Other Supplementary Materials for this manuscript include the following:**

Not applicable

**RESOURCES TABLE**

| **REAGENT or RESOURCE** | **SOURCE** | **IDENTIFIER** |
| --- | --- | --- |
| Hoechst 33258 | Beyotime | C1011 |
| Coomassie Brilliant Blue R250 | Beyotime | ST031 |
| NaCl | Sigma | S3014 |
| Tris-HCl | Sigma | 10812846001 |
| MgCl_2_ | Sigma | M8266 |
| EDTA | Sigma | E6758 |
| Acetone | Sigma | 67-64-1 |
| Dithiothreitol | Solarbio | R0861 |
| Acetonitrile | Sigma | 75-05-8 |
| Formic acid | Sigma | 64-18-6 |
| Ethanol | Sigma | 64-17-5 |
| Acetic acid | Sigma | 64-19-7 |
| Triton-X-100 | Biosharp | BS084 |
| BSA | Sigma | A1933 |
| Biotin-FITC | Thermo Scientific | 22030 |
| Biotin-Cy5 | Xi’an Qiyue  Biology | Q-0255871 |
| Biotin-PE | Xi’an Qiyue  Biology | Customized |
| Kanamycin | Sigma | 420311 |
| ACK Lysis Buffer | Gibco | A1049201 |
| IPTG | Sigma | I6758 |
| Ni-Charged Resin | Genscript | L00666 |
| SDS | Beyotime | ST627 |
| PBS | Gibco | 10010023 |
| DMSO | Sigma | D8418 |
| CA630 | Sigma | I3021 |
| Tween-20 | Sigma | 11332465001 |
| Digitonin | Invitrogen | BN2006 |
| RNase Inhibitor | Invitrogen | AM2682 |
| Glycerol | Sangon | A100854 |
| Nuclease-free Water (NF-H_2_O) | Thermo Scientific | 10977015 |
| SPRIselect beads | Beckman Coulter | B23317 |
| MobiCube® 3’ RNA-Seq  Reverse Transcription Kit | Mobidrop | PN-S050500301 |
| Mobicube® 3’ RNA-Seq Gel Beads Kit | Mobidrop | PN-S050400301 |
| MobiCube® 3’ RNA-Seq Library Reagent Kit | Mobidrop | PN-S050600301 |
| MobiCube® 3’ RNA-Seq Chip A  Single Cell Kit | Mobidrop | PN-S050100301 |
| Mobicube® 3’ RNA-Seq Dual Index Kit | Mobidrop | PN-S050300301 |
| Qubit ssDNA HS Quantitation Kit | Invitrogen | Q10212 |
| Qubit dsDNA HS Quantitation Kit | Invitrogen | Q32854 |
| Barcode (Oligo) | Sangon | Customized |
| Barcode Pre-amplification Primer | Sangon | Customized |
| **Software** |  |  |
| ImageJ | NIH, Bethesda | https://imagej.nih.gov/ij/ |
| Origin 9.0 | OriginLab | https://www.mediacy.com/imageproplus |
| Image Pro-Plus 6.0 | MediaCybernetics® | https://www.originlab.com |
| CytExpert | Beckman Coulter | https://www.beckman.com/flow-cytometry/software |
| FlowJo | BD Biosciences | https://www.flowjo.com/ |
| Cellranger | 10X Genomics | https://www.10xgenomics.com/support/software/cell-ranger/latest |
| Scanpy toolkit | Helmholtz Center Munich, Theislab | Scanpy toolkit |

**Materials and Methods**

**Cell Culture**

H1975 cells were cultured at 37°C with 5% CO2 in RPMI 1640 complete medium (GIBCO), supplemented with 10% fetal bovine serum (FBS) (Every Green, Zhejiang Tianhang Biotechnology Co., Ltd.) and 1% penicillin-streptomycin (GIBCO). NIH 3T3, MDA-MB-231, MCF-7, and HEK 293T cells were maintained under the same temperature and CO2 conditions in Dulbecco’s Modified Eagle Medium (DMEM, GIBCO), similarly supplemented with 10% FBS and 1% penicillin-streptomycin. Prior to experiments, all cells were cultured in 25 cm² tissue culture dishes (LabServ).

**Analysis of Captured N-glycan**

The released N-glycans from RNase B were purified and analyzed following a previously reported method ^[1]^. The dried N-glycans were reconstituted in PBS and incubated with 0.5 mg of Stv-Fg protein at 4°C for 1 hour. The mixtures were then transferred to an Amicon® Ultra 10 kDa MWCO filter and centrifuged at 5,000 g for 10 minutes at 4°C. The mixtures were washed four times with 200 μL of 50 mM NH_4_HCO_3_, and the captured glycans were eluted from the Stv-Fg protein with 100 μL of 50% formic acid, repeated three times. Finally, the eluted glycans were dried using a SpeedVac to remove residual formic acid and NH_4_HCO_3_, preparing them for analysis.

**Confocal Laser Scanning Microscopy**

After staining the cells or nuclei with different dyes, the buffer was removed and replaced with fresh PBS three times to prepare for confocal laser scanning microscopy. All images were acquired using a Fluoview FV3000 confocal laser scanning microscope equipped with an IX83 fully motorized inverted microscope and a 100× UPLSAPO oil immersion lens (numerical aperture 1.4) (Olympus), as described previously ^[2]^. Hoechst 33258, Biotin-FITC, or Dil were excited at 405, 488, or 561 nm, respectively, and detected at 430-470 nm, 491-535 nm, or 570-670 nm. Image analysis was performed using ImageJ software (<https://imagej.en.softonic.com/>).

**Fluorescent Stv-Fg Preparation**

In a dark environment, 5 μL of Stv-Fg solution (6.25 μM) was added to 90 μL of PBS buffer. Then, 5 μL of either Biotin-Cy5 solution (14.7 μM) or Biotin-PE solution (14 μM) was added and mixed thoroughly. The mixture was incubated at 4°C for 20 minutes, resulting in a Stv-Fg-Cy5 or Stv-Fg-PE solution with an approximate concentration of 6.25 μM. This solution should be used immediately or temporarily stored at 4°C in the dark.

**Viability and concentration quantification** (via AO/PI staining and Countstar Mira FL analysis) **and temporary ice storage** mirrored the nucleus barcoding protocol.

**Library Preparation**

MobiCube® 3’ RNA-seq kits were used, following the user manual for MobiCube® high-throughput single-cell 3' transcriptome reagents up to the cDNA pre-amplification step. This included reagents preparation, chip preparation, sample loading with MobiNova-100 (MobiDrop), droplet pre-treatment with the MobiDrop droplet pre-treatment device, droplet reverse transcription using the MiniAmp Plus PCR machine (Thermo Fisher), emulsion breakage, filtration and washing with the MobiCube® filter, cDNA cleanup with SPRIselect beads, and pre-amplification with the MiniAmp Plus PCR machine.

Pre-amplified cDNA Cleanup: 60 μL (0.6×) of SPRIselect beads were added to a PCR tube (Sarstedt) containing the pre-amplified cDNA and gently mixed using a vortex mixer. The mixture was incubated at room temperature for 5 minutes. After incubation, the tube was briefly centrifuged and placed on a 0.2 mL magnetic stand, allowing the solution to clarify for approximately 3 minutes. 150 μL of the supernatant were then transferred to a new 200 μL centrifuge tube for temporary storage. 200 μL of freshly prepared 80% ethanol was added to the tube along the wall, avoiding contact with the beads, and allowed to stand for 30 seconds.

Subsequent Operations for Endogenous Transcript: The supernatant was discarded, and 200 μL of freshly prepared 80% ethanol was added to the tube along the wall without touching the beads. After standing for 30 seconds, the supernatant was discarded, and the tube was briefly centrifuged with the bead-containing side facing outward. The PCR tube was then placed on a magnetic stand for 10 seconds. Any remaining 80% ethanol was removed with a 20 μL pipette, repeating the process as necessary until no visible liquid remained. The PCR tube was left open to air dry the beads for approximately 3-5 minutes, ensuring no water reflection on the bead surface while avoiding excessive drying that could cause bead cracking. The PCR tube was then removed from the magnetic stand, and 25 μL of DNA elution buffer was added. The pipette volume was adjusted to 18 μL, and the beads were thoroughly mixed with the liquid by pipetting up and down 15 times. The mixture was incubated at room temperature for 3 minutes. After incubation, the tube was briefly centrifuged and placed back on the magnetic stand to allow the solution to clarify for approximately 3 minutes. 23 μL of the eluate was transferred to a new 0.2 mL PCR tube.

Subsequent Operations for Barcode cDNA: 75 μL of the supernatant from the preceding step were combined with 70 μL (2.1×) of SPRIselect beads and gently mixed using a vortex mixer. The mixture was incubated at room temperature for 5 minutes. Following incubation, the tube was briefly centrifuged and placed on a 0.2 mL magnetic stand to allow the solution to clarify, which took approximately 3 minutes. The supernatant was discarded, and 200 μL of freshly prepared 80% ethanol was added to the PCR tube along the wall without touching the beads, letting it stand for 30 seconds. After discarding the supernatant, this step was repeated. The supernatant was discarded again, and the tube was briefly centrifuged with the bead-containing side facing outward before being placed on the magnetic stand for 10 seconds. Any remaining 80% ethanol was removed using a 20 μL pipette, repeating the process as needed until no visible liquid remained. The PCR tube was left open to air dry the beads for approximately 3-5 minutes, ensuring no water reflection on the bead surface while avoiding excessive drying that could cause bead cracking. The PCR tube was then removed from the magnetic stand, and 22 μL of DNA elution buffer was immediately added. The pipette volume was adjusted to 18 μL, and the beads were thoroughly mixed with the liquid by pipetting up and down 15 times. The mixture was incubated at room temperature for 3 minutes. After incubation, the tube was briefly centrifuged, then placed back on the magnetic stand to allow the solution to clarify for approximately 3 minutes. Finally, 20 μL of the eluate was transferred to a new 0.2 mL PCR tube for subsequent barcode library amplification.

Stopping Point: The sample can be stored at 4°C for up to 72 hours, at -20°C for up to 4 weeks, or you may proceed to the next step in the experiment.

cDNA Quality Control: 1 μL of the purified cDNA sample was used to measure the concentration with a Qubit instrument. Additionally, 1 μL of the purified cDNA sample was taken for quality analysis using the Agilent 2100 HS DNA analyzer.

**Transcriptome Library Construction**

cDNA Fragmentation: The fragmentation buffer was fully thawed, and both the fragmentation enzyme and buffer were vortexed for 5-8 seconds, followed by brief centrifugation and placement on ice. The amount of cDNA required for the fragmentation reaction was determined according to the MobiCube® Single-Cell 3’ RNA-seq Kit User Guide for each sample, with any remaining cDNA stored at -20°C. The total volume of the fragmentation reaction was adjusted to 35 μL. If the sample volume was less than 35 μL, NF-H_2_O was added to reach the final volume. The fragmentation reagent was prepared on ice, consisting of 5 μL of fragmentation buffer and 10 μL of fragmentation enzyme, which were vortexed thoroughly to mix. Then, 15 μL of this fragmentation reagent was added to each sample tube on ice, mixed slowly by pipetting up and down, and briefly centrifuged. The PCR machine was pre-set with the following program: thermal lid temperature set to 75°C, reaction volume of 50 μL, with the following steps: 4°C hold, 32°C for 8 minutes, 65°C for 30 minutes, and an 8°C hold. After completing the reaction, the sample tubes were immediately placed on ice, and the process continued to the next step.

Adapter Ligation: The ligation reaction reagent was prepared on ice for each tube, consisting of 20 μL of ligation buffer, 10 μL of ligation enzyme, and 16 μL of NF-H_2_O. The ligation buffer was mixed by pipetting up and down 15 times, and the ligation enzyme was inverted 5 times by flicking with fingers. Forty-six microliters of this ligation reaction reagent were added to each sample tube on ice, followed by thorough mixing using a vortex and brief centrifugation. 4 μL of adapter was then added to each sample tube. The contents were mixed thoroughly by pipetting up and down 15 times and briefly centrifuged. The PCR machine was set with the following program: 20°C for 15 minutes, then a 4°C hold, with the thermal lid turned off and a reaction volume of 100 μL. After completing the reaction, the process immediately proceeded to the next step.

Post-Ligation Cleanup: The SPRIselect beads were thoroughly vortexed before use. Forty microliters of NF-H_2_O were added to the PCR tube containing the ligation products, followed by the addition of 42 μL (0.3×) of SPRIselect beads. The mixture was vortexed 10 times, incubated at room temperature for 5 minutes, then briefly centrifuged. The tube was placed on a 0.2 mL magnetic rack for 5 minutes until the solution became clear. A pipette was set to 175 μL to transfer the supernatant to a new 0.2 mL PCR tube, discarding the beads. Forty-two microliters (0.3×) of SPRIselect beads were added to the new PCR tube, vortexed 10 times to mix, and incubated at room temperature for 5 minutes. After briefly centrifuging, the PCR tube was placed on the magnetic rack until the solution became clear. The supernatant was carefully discarded, avoiding the beads. While keeping the PCR tube on the magnetic rack, 200 μL of freshly prepared 80% ethanol was added along the side of the tube, avoiding the beads, and allowed to stand for 30 seconds. The supernatant was discarded, and the step was repeated once more. After discarding the supernatant again, the tube was briefly centrifuged (with the beads facing outward) and placed back on the magnetic rack for 10 seconds. Any remaining 80% ethanol was removed using a 20 μL pipette until no visible liquid remained. The tube cap was opened, and the beads were air-dried for 2-3 minutes until no visible moisture remained, taking care to avoid over-drying to prevent bead cracking. The tube was immediately removed from the magnetic rack, and 22 μL of NF-H_2_O was added to each tube. A 20 μL pipette was set to 15 μL and used to pipette up and down 15 times until the beads were fully resuspended in the liquid. The mixture was incubated at room temperature for 3 minutes, briefly centrifuged, and the tube was placed back on the magnetic rack until the solution became clear. Finally, 20 μL of the elution buffer was transferred to a new 0.2 mL PCR tube and placed on ice.

Library Indexing and Amplification: On ice, 25 μL of amplification mix was added to the sample tube containing 20 μL of purified ligation products, followed by the addition of 5 μL of 3' dual index to the 45 μL reaction mixture. The 3' dual index number for each sample was recorded to avoid cross-contamination. The mixture was then pipetted up and down 15 times to mix slowly, avoiding the formation of bubbles. PCR amplification was performed with the following program: 98°C for 2 minutes, 98°C for 20 seconds, 65°C for 30 seconds, 72°C for 30 minutes, repeating steps 98°C for 20 seconds, 65°C for 30 seconds, and 72°C for 30 minutes for 9-16 cycles, followed by 72°C for 1 minute, and finally held at 8°C.

Stopping Point: The sample can be stored at 4°C for up to 72 hours or proceed to the next step in the experiment.

Library Fragment Selection: The SPRIselect beads were vortexed thoroughly to mix before use. Then, 50 μL of DNA Elution Buffer was added to the PCR tube containing the library amplification products and mixed by pipetting up and down. Next, 50 μL (0.5×) of SPRIselect beads was added to the PCR tube, and the mixture was vortexed 10 times to mix. After incubating at room temperature for 5 minutes, the PCR tube was briefly centrifuged and placed on a 0.2 mL magnetic rack until the solution was clear, approximately 3 minutes. A pipette was set to 145 μL to transfer the supernatant to a new 0.2 mL PCR tube, discarding the beads. Then, 25 μL (0.25×) of SPRIselect beads was added to the new PCR tube and vortexed 10 times to mix. The mixture was incubated at room temperature for 5 minutes, briefly centrifuged, and placed on the magnetic rack until the solution was clear, approximately 3 minutes. The supernatant was carefully discarded, avoiding the beads. While keeping the PCR tube on the magnetic rack, 200 μL of freshly prepared 80% ethanol was added along the side of the tube, avoiding the beads, and the supernatant was discarded. This step was repeated once more. The tube was briefly centrifuged with the beads facing outward, placed back on the magnetic rack for 10 seconds, and any remaining 80% ethanol was removed using a 20 μL pipette until no visible liquid remained. The PCR tube cap was opened and the beads were air-dried for 1-2 minutes until no visible liquid remained on the beads, taking care to avoid over-drying to prevent cracking. The tube was then removed from the magnetic rack, and 22 μL of DNA elution buffer was immediately added. A 20 μL pipette was set to 20 μL, and the mixture was pipetted up and down 15 times to ensure the beads were fully resuspended in the liquid. After incubating at room temperature for 3 minutes, the tube was briefly centrifuged and placed back on the magnetic rack until the solution was clear, approximately 3 minutes. Finally, 20 μL of the elution buffer was transferred to a new 0.2 mL PCR tube.

Stopping Point: The sample can be stored at 4°C for up to 72 hours or at -20°C for long-term storage.

Library Quality Control: 1 μL of the purified library product was taken for Qubit concentration measurement. Based on the measured concentration, another 1 μL of the sample was taken and diluted as needed for quality analysis using the Agilent 2100 HS DNA analyzer. Qualified libraries were then selected for the next step of sequencing.

**Barcode Library Construction**

Barcode Library Amplification: Five microliters of purified barcode cDNA (from the "Pre-amplified cDNA cleanup" section) were used for barcode amplification. On ice, 25 μL of amplification mix was added to the 5 μL of barcode cDNA. Additionally, 2.5 μL of dual index reagent was added to each sample, with the dual index number recorded to avoid cross-contamination. Seventeen and a half microliters of NF-H₂O were then added, and the mixture was gently mixed by pipetting up and down 15 times to avoid bubble formation. The following PCR program was used for amplification: 98°C for 30 seconds, followed by 98°C for 10 seconds and 65°C for 75 seconds, repeated 12 times, then 65°C for 5 minutes, and finally held at 8°C.

Barcode Library Fragment Purification: The SPRIselect beads were thoroughly vortexed before use. Sixty microliters (1.2×) of SPRIselect beads were added to the PCR tube containing the barcode library amplification products. This mixture was vortexed 10 times and incubated at room temperature for 5 minutes. After incubation, the tube was briefly centrifuged and placed on a 0.2 mL magnetic rack until the solution clarified, which took approximately 3 minutes. The supernatant was carefully discarded, avoiding the beads. While the tube remained on the magnetic rack, 200 μL of freshly prepared 80% ethanol was added along the side of the tube, avoiding contact with the beads, and the supernatant was discarded. This ethanol wash step was repeated once more. The tube was briefly centrifuged with the beads facing outward, then placed back on the magnetic rack for 10 seconds. Any remaining 80% ethanol was removed using a 20 μL pipette, with the process repeated until no visible liquid remained. The PCR tube cap was then opened to air-dry the beads for 1-2 minutes until no visible moisture remained, taking care not to over-dry to prevent bead cracking. After drying, 22 μL of DNA elution buffer was immediately added to the tube. A 20 μL pipette was set to 20 μL and used to pipette up and down 15 times to ensure the beads were fully resuspended in the liquid. The mixture was incubated at room temperature for 3 minutes. The tube was then briefly centrifuged and placed back on the magnetic rack until the solution was clear, which took approximately 3 minutes. Finally, 20 μL of the elution buffer was transferred to a new 0.2 mL PCR tube.

Stopping Point: The sample can be stored at 4°C for up to 72 hours or at -20°C for long-term storage.

Barcode Library Quality Control: One microliter of the purified barcode library product was used for Qubit concentration measurement. Based on this concentration, the sample was diluted as needed for Qsep100 quality control analysis. Qualified libraries were then selected for the next step of sequencing.

Sequencing: Both the barcode library and endogenous cDNA library should be sequenced separately. The sequencing platform used was NovaSeq S4, with sequencing type set to PE150.

**Bioinformatic Analysis**

The barcode-filtered gene expression matrix was generated using Cellranger (https://www.10xgenomics.com/support/software/cell-ranger/latest). We conducted scRNA-seq/snRNA-seq data analysis and visualization using the Scanpy toolkit (https://scanpy.readthedocs.io/en/stable/), covering preprocessing, integration, visualization, clustering, and cell type identification. To mitigate biases from variations in sequencing depth, we normalized each cell's total gene expression to 10,000 using Scanpy's ‘pp.normalize_total’ function. The normalized datasets were then used to construct a shared nearest neighbor (SNN) graph. Principal component analysis (PCA) was applied, and the ‘sc.pp.neighbors’ function was used with 15 neighbors and 30 principal components. Clustering was performed using the ‘sc.tl.leiden’ function with resolutions ranging from 0.1 to 1. Clusters were visualized using Uniform Manifold Approximation and Projection (UMAP) or t-distributed Stochastic Neighbor Embedding (t-SNE) based on the principal components. Cell-type identities for each cluster were manually assigned using a published list of marker genes.

**Statistical analyses**All data were presented as mean ± SD. Unpaired student’s *t* test was used for comparisons between two groups. All statistical analyses were performed in the Origin 9.0 software. **p*<0.05, ***p*<0.01, ****p*<0.001, respectively.

**Data availability**

All primary data related to this study are included in the Source Data files accompanying each figure in this paper. Additional data supporting the findings are available from the corresponding author upon reasonable request.

**Table S1. Barcode Sequence and Barcode Pre-amplification Primer Sequence**

|  | **Name** | **Sequence (5' to 3')** | **5' end modification** |
| --- | --- | --- | --- |
| **Barcode** | Tag 1 | GTCTCGTGGGCTCGGAGATGTGTATAAGAGACAGATACCAATACCAGCABAAAAAAAAAAAAAAAAAAAAAAAAAAAAAA | Biotin |
|  | Tag 2 | GTCTCGTGGGCTCGGAGATGTGTATAAGAGACAGGCTCAAGCTCAAGCTBAAAAAAAAAAAAAAAAAAAAAAAAAAAAAA | Biotin |
|  | Tag 3 | GTCTCGTGGGCTCGGAGATGTGTATAAGAGACAGCATCGCCATCGCCATBAAAAAAAAAAAAAAAAAAAAAAAAAAAAAA | Biotin |
|  | Tag 4 | GTCTCGTGGGCTCGGAGATGTGTATAAGAGACAGCTGTGACTGTGACTGBAAAAAAAAAAAAAAAAAAAAAAAAAAAAAA | Biotin |
|  | Tag 5 | GTCTCGTGGGCTCGGAGATGTGTATAAGAGACAGGGACATGGACATGGABAAAAAAAAAAAAAAAAAAAAAAAAAAAAAA | Biotin |
|  | Tag 6 | GTCTCGTGGGCTCGGAGATGTGTATAAGAGACAGCAACGTCAACGTCAABAAAAAAAAAAAAAAAAAAAAAAAAAAAAAA | Biotin |
|  | Tag 7 | GTCTCGTGGGCTCGGAGATGTGTATAAGAGACAGAAGCGTAAGCGTAAGBAAAAAAAAAAAAAAAAAAAAAAAAAAAAAA | Biotin |
|  | Tag 8 | GTCTCGTGGGCTCGGAGATGTGTATAAGAGACAGAATAGCAATAGCAATBAAAAAAAAAAAAAAAAAAAAAAAAAAAAAA | Biotin |
|  | Tag 9 | GTCTCGTGGGCTCGGAGATGTGTATAAGAGACAGCCAGCATTCAGCATCBAAAAAAAAAAAAAAAAAAAAAAAAAAAAAA | Biotin |
|  | Tag 10 | GTCTCGTGGGCTCGGAGATGTGTATAAGAGACAGGAGCATGAGCATGAGBAAAAAAAAAAAAAAAAAAAAAAAAAAAAAA | Biotin |
|  | Tag 11 | GTCTCGTGGGCTCGGAGATGTGTATAAGAGACAGGGTACAGGTACAGGTBAAAAAAAAAAAAAAAAAAAAAAAAAAAAAA | Biotin |
|  | Tag 12 | GTCTCGTGGGCTCGGAGATGTGTATAAGAGACAGTTGGACTTGGACTTGBAAAAAAAAAAAAAAAAAAAAAAAAAAAAAA | Biotin |
|  | Tag 13 | GTCTCGTGGGCTCGGAGATGTGTATAAGAGACAGAAGTATGCCCTACGABAAAAAAAAAAAAAAAAAAAAAAAAAAAAAA | Biotin |
|  | Tag 14 | GTCTCGTGGGCTCGGAGATGTGTATAAGAGACAGCACCGTCATTCAACCBAAAAAAAAAAAAAAAAAAAAAAAAAAAAAA | Biotin |
|  | Tag 15 | GTCTCGTGGGCTCGGAGATGTGTATAAGAGACAGCTCAGATGCCCTTTABAAAAAAAAAAAAAAAAAAAAAAAAAAAAAA | Biotin |
|  | Tag 16 | GTCTCGTGGGCTCGGAGATGTGTATAAGAGACAGAGACTAATAGCTGACBAAAAAAAAAAAAAAAAAAAAAAAAAAAAAA | Biotin |
| **Barcode Pre-amplifi-cation Primer** | Barcode  Pre-amplifi-cation  Primer | GTCTCGTGGGCTCGGAGATGTGTATAA | N/A |

**Table S2. Overall Classification Accuracy (OCA) of Lipid-, Antibody-, and Stv-Fg-Based**

| Metric | Antibodies | Lipids | Stv-Fg |
| --- | --- | --- | --- |
| OCA (Cells) | 0.91-0.96 | 0.68–0.84 | 0.969 |
| OCA (Nuclei) | 0.51 | 0.84 | 0.987 |

Figure S1

**
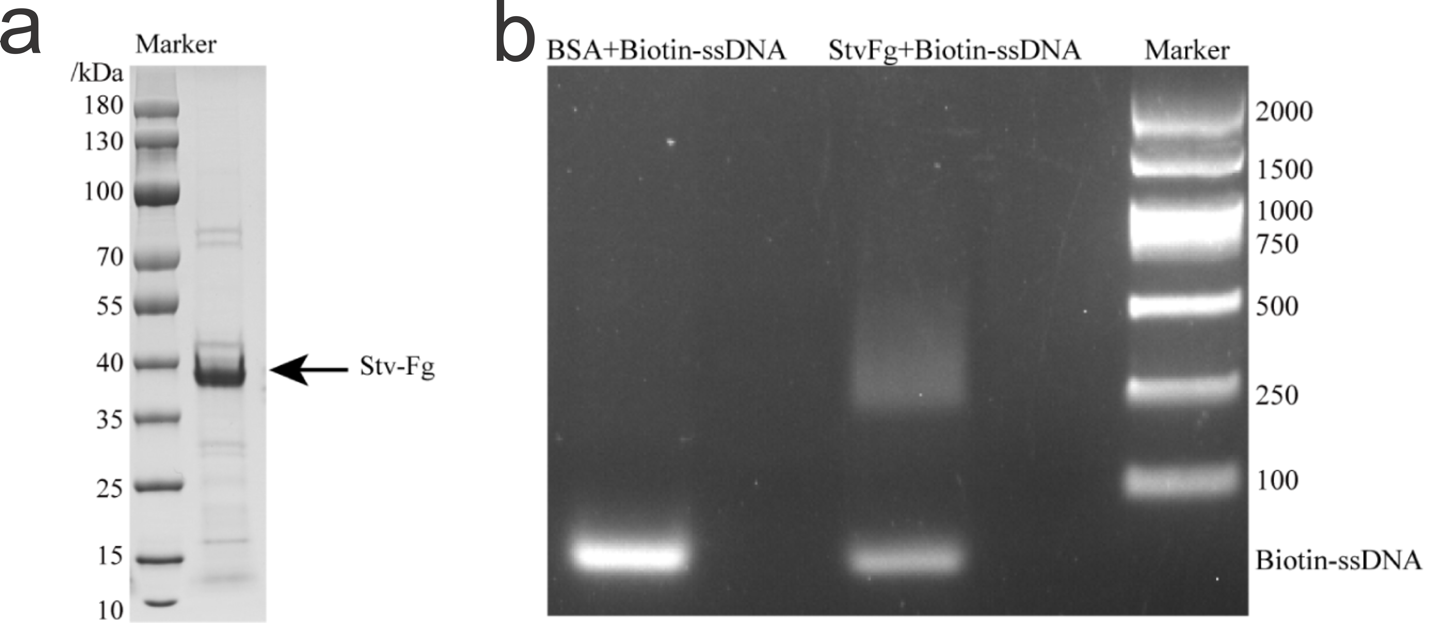
**

**Fig. S1. Construction of Stv-Fg fusion protein and toti-N-seq DNA barcode (Stv-Fg-ssDNA). a,** Purification of Stv-Fg fusion protein. **c,** Purification of toti-N-seq DNA barcode (Stv-Fg-ssDNA).

Figure S2

**
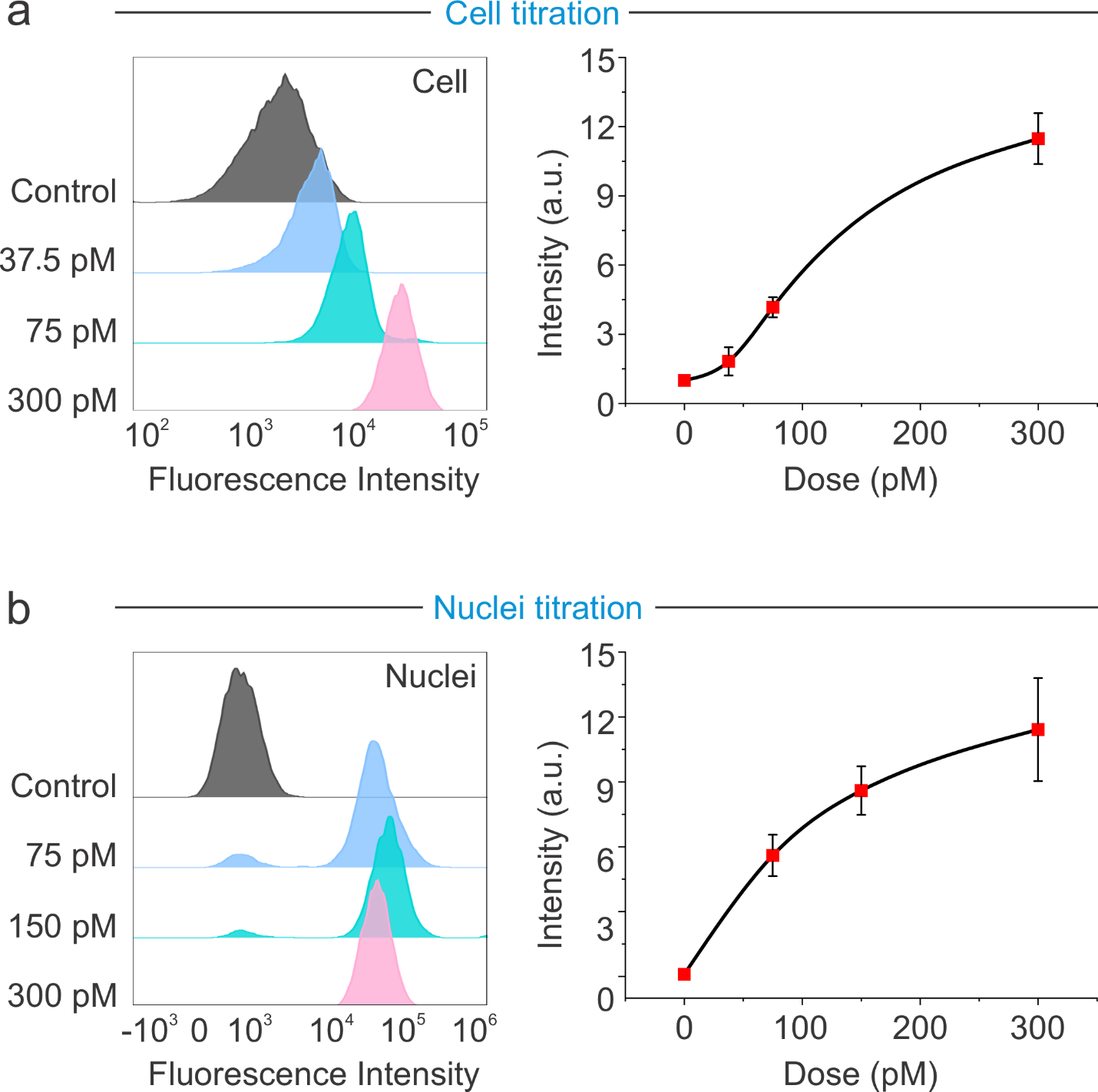
**

**Fig. S2. Titration assay for toti-N-seq fluorescent barcode for single cell labelling (a) and single nucleus labelling respectively (b).**

Figure S3

**
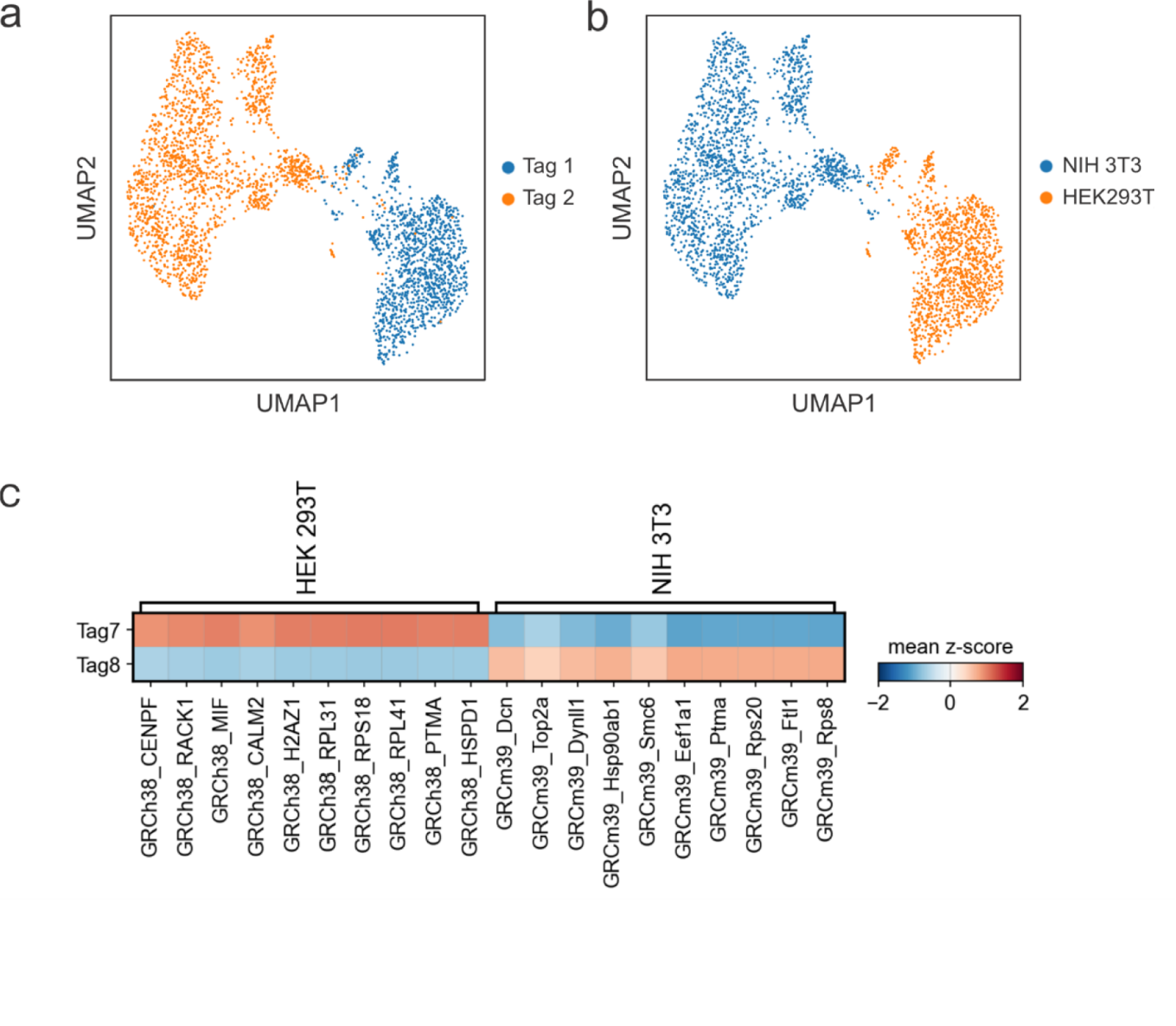
**

**Fig. S3. Toti-N-glycan barcodes for sc-seq of pooled samples. a-b,** Two sampled cells from two cell types were clustered based on their gene expression profiles. Each individual cells was indicated by their corresponding tags (a) and cell types (b). **c,** Tags of cells exhibit different DEGs.

Figure S4


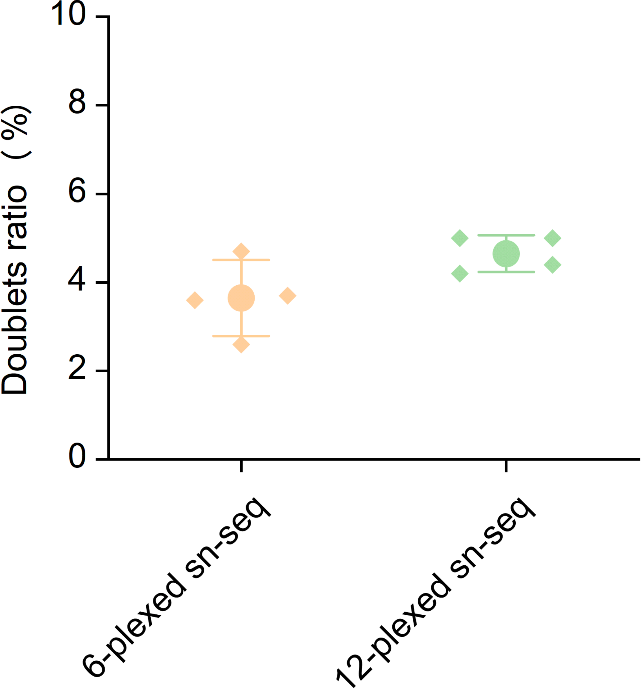


**Fig. S4. The ratio of doublets obtained by our toti-N-multiplex sn-seq with 6-plexed capacity and 12-plexed capacity respectively.**

Figure S5

**
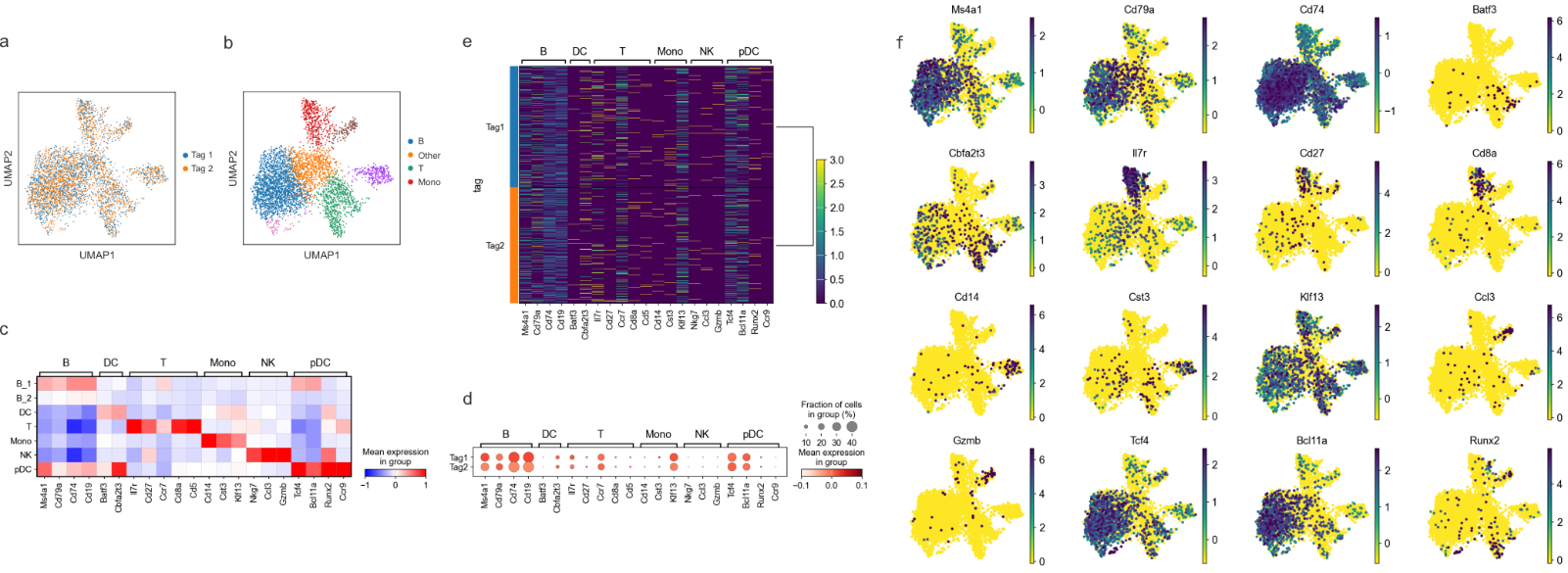
**

**Fig. S5. Toti-N-sn-seq demultiplexed mouse PBMCs. a-b,** Two sampled nuclei from mouse PBMCs were clustered based on their gene expression profiles. Each individual nucleus was indicated by their corresponding tags (a) and cell types (b). **c,** Two tagged samples of nuclei exhibited different signature marker genes. **d,** Groups of nuclei from different cell types in mouse PBMCs exhibited different signature marker genes. e, Heatmap showed nuclei with different tags expressing different signature marker genes. f, Expression of signature marker genes of individual nuclei from mouse PBMCs in gene expression space.

Figure S6

**
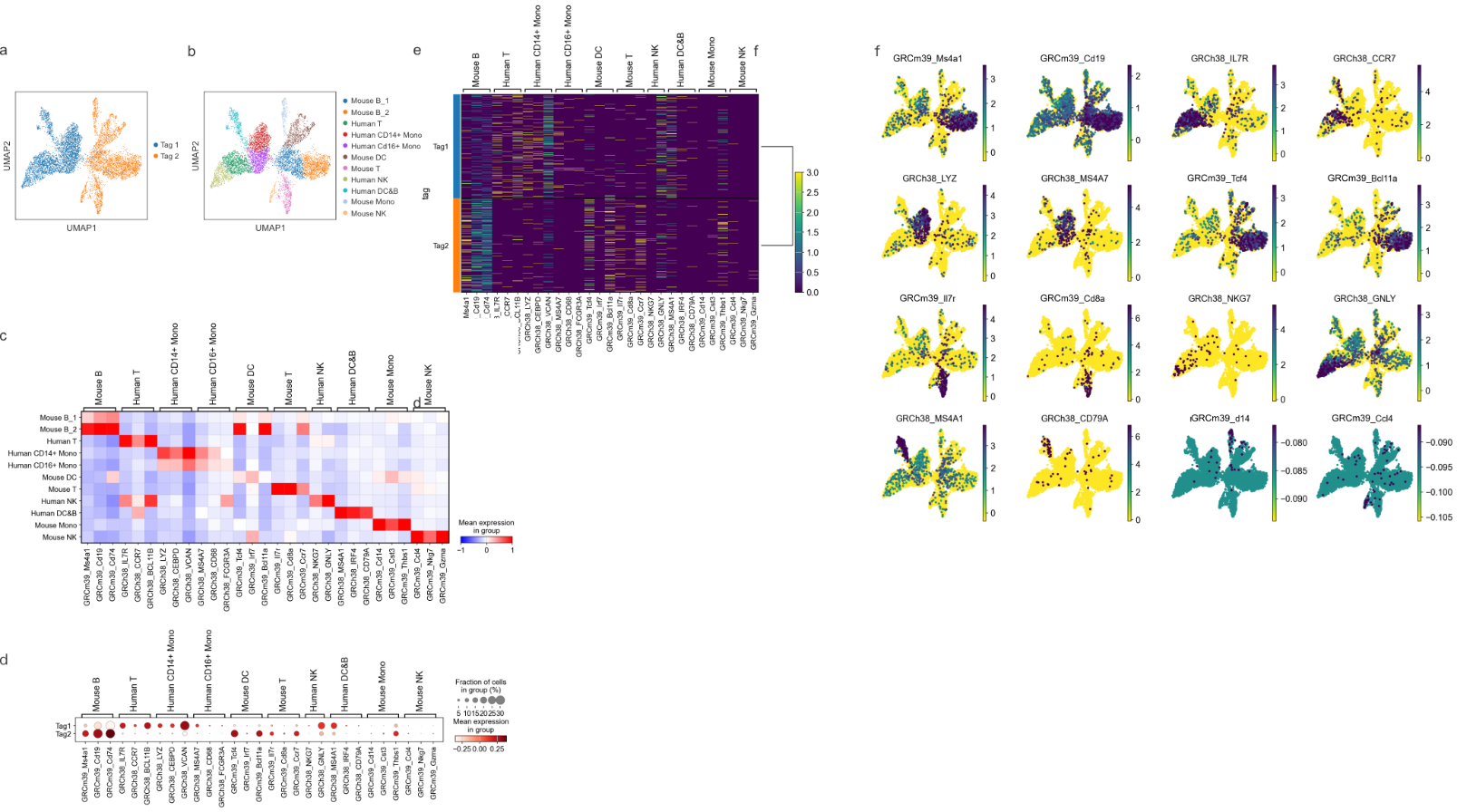
**

**Fig. S6. Toti-N-sn-seq demultiplexed mixed human and mouse PBMCs. a-b,** Two sampled nuclei from mixed human and mouse PBMCs were clustered based on their gene expression profiles. Each individual nucleus was indicated by their corresponding tags (a) and cell types (b). **c,** Two tagged samples of nuclei exhibited different signature marker genes. **d,** Groups of nuclei from different cell types in mixed human and mouse PBMCs exhibited different signature marker genes. e, Heatmap showed nuclei with different tags expressing different signature marker genes. f, Expression of signature marker genes of individual nuclei from mixed human and mouse PBMCs in gene expression space.

**References**

[1] Zhang L, Wang W, Yang Y, Zhu W, Li P, Wang S, Liu X. Site-specific, covalent immobilization of PNGase F on magnetic particles mediated by microbial transglutaminase. Anal Chim Acta. 2023 Apr 15;1250:340972. doi: 10.1016/j.aca.2023.340972.

[2] Li P, Chen P, Qi F, Shi J, Zhu W, Li J, Zhang P, Xie H, Li L, Lei M, Ren X, Wang W, Zhang L, Xiang X, Zhang Y, Gao Z, Feng X, Du W, Liu X, Xia L, Liu BF, Li Y. High-throughput and proteome-wide discovery of endogenous biomolecular condensates. Nat Chem. 2024 Mar 18. doi: 10.1038/s41557-024-01485-1.
